# Potential association between arsenic and vitamin D

**DOI:** 10.3389/fendo.2024.1430980

**Published:** 2024-07-17

**Authors:** Mythri Chittilla, Chantal Uzoma, Desiree Brewer, Mohammed S. Razzaque

**Affiliations:** ^1^ Department of Pathology, Lake Erie College of Osteopathic Medicine (LECOM), Erie, PA, United States; ^2^ Department of Medical Education, School of Medicine, University of Texas Rio Grande Valley (UTRGV), Edinburg, TX, United States

**Keywords:** arsenic, vitamin D, skin, tumor, T cell

## Introduction

Vitamin D is a pleiotropic hormone that regulates calcium and phosphate homeostasis. The roles of vitamin D can be divided into skeletal and nonskeletal. Its skeletal roles include promoting calcium absorption in the intestines, maintaining adequate levels of calcium and phosphate in the bloodstream, and supporting bone mineralization, thus preventing conditions such as rickets and osteoporosis. Nonskeletal roles encompass a range of functions, including immune system modulation, cardiovascular health, and cell growth regulation. Sufficient concentrations of vitamin D in the bloodstream are 20–100 ng/ml in men, women, and children, but individuals with more pigmented skin, live in environments with less sun exposure, or who are older in age are at increased risk of vitamin D deficiency (1). In the past decade, vitamin D has been repeatedly shown to halt tumorigenesis, positively affect T-cell function, improve lung function, and play a role in the pathogenesis of diabetes mellitus. Arsenic has been shown to have harmful consequences on human health, and more individuals are exposed to increasing amounts of arsenic in their environment due to natural sources, such as mineral deposits in water, and unnatural sources, such as pollution. This brief article describes how arsenic might interact with vitamin D.

## Arsenic toxicity

Chronic and acute exposure to arsenic causes various harmful consequences. According to the CDC, more than 60,000 ppb of arsenic in water can result in death. Arsenic is naturally found in the environment and enters water via the erosion of rocks that contain this element, or it can be incorporated into food and water via pollution (2). In China, rice contains high concentrations of arsenic, with an average consumption of 3.52 × 10^−4^ mg/kg of body weight per day, due to the soil (3). Different forms of arsenic include inorganic arsenic, monomethylarsonic acid, dimethylarsinic acid, arsenobetaine, and arsenic trioxide (As_2_O_3_). Short-term and long-term exposure to arsenic can have significant health implications, primarily through the ingestion of contaminated water, food, or air. In the short term, high levels of arsenic exposure can lead to acute symptoms such as abdominal pain, vomiting, diarrhea, and, in severe cases, cardiovascular and neurological issues (2). Specifically, acute ingestion of large quantities of arsenic can cause gastrointestinal symptoms within hours and can lead to bloody diarrhea, resulting in hypovolemic shock. Acute cardiovascular effects include QT interval prolongation, arrythmias, and tachycardia. Serious neurological issues include seizures and coma (2). On the other hand, long-term exposure to lower levels of arsenic, often through chronic ingestion of contaminated water, has been linked to more insidious health issues (2). Prolonged exposure is associated with an increased risk of various chronic conditions, including skin lesions (hyperpigmentation and hyperkeratosis), cardiovascular diseases (coronary heart disease, peripheral vascular disease, endothelial dysfunction, hypertension, and atherosclerosis), respiratory problems (chronic obstructive pulmonary disease), neurological conditions (peripheral neuropathy) and certain cancers, such as lung and bladder cancer (2, 4). Management of arsenic toxicity primarily involves removal of the element from further exposure, supportive care to address symptoms such as fluid and electrolyte imbalance, and specific therapies such as chelation therapy to enhance the elimination of arsenic from the body. Chelation therapy includes dimercaprol and dimercaptosuccinic acid. Long-term monitoring and follow-up are crucial for assessing and managing any lingering effects or complications. Notably, chronic arsenic toxicity poses a more subtle but pervasive threat to public health, emphasizing the importance of addressing arsenic contamination in environmental sources to prevent long-term adverse health consequences.

## Vitamin D, arsenic, and tumorigenesis

Numerous studies have linked vitamin D inadequacy to increased incidences of colon, prostate, breast, and several hematological cancers. The most compelling evidence shows a strong relationship between vitamin D and colorectal cancer and hematological cancers. To a lesser extent, data show that prostate, breast, and skin cancers are also affected by vitamin D. In colon carcinoma cells, the vitamin D and vitamin D receptor (VDR) complex binds to β-catenin and is associated with complexes within the nucleus, stopping cell proliferation (5, 6). The vitamin D and VRD complex has also been shown to have antiproliferative effects on colorectal cancer by directly and indirectly repressing the WNT/β-catenin pathway, which is most active in colorectal cancer (7). Most importantly, a large portion of advanced colorectal cancer patients lack expression of the VRD gene SNAI1 (snail family transcriptional repressor 1) and SNAI2 (snail family transcriptional repressor 2) proteins, which directly bind and block transcription of the VDR promoter (8, 9), and supplementing these patients with vitamin D in the early stage of colorectal cancer is associated with positive outcomes but not in the advanced stages (5).

Vitamin D deficiency has also been shown to be significantly related to hematological and skin cancer. Vitamin D plays an important role in hematopoiesis and differentiation into myelocytes and granulocytes, hence causing uncontrolled proliferation in undifferentiated neoplastic cells, such as in acute myelocytic leukemia (AML). Vitamin D deficiency has been linked to poorer prognosis in patients with hematological malignancies by limiting differentiation, halting apoptosis, and sensitizing neoplastic cells to anticancer treatments (10, 11). Numerous studies have demonstrated that 1,25(OH)_2_D_3_ decreases the phosphorylation of the Janus kinase (JAK) and STAT signaling pathways, both of which are the most commonly overactive pathways in leukemia and lymphoma (12, 13). It is difficult to determine whether vitamin D has preventative effects on skin cancer since UV light is used to convert vitamin D to its active form but can also induce DNA damage. In squamous cell carcinoma, vitamin D and topical cholecalciferol supplementation has been shown to significantly delay the progression of this cancer (14, 15). Similarly, insufficient serum vitamin D levels have been shown to lead to a worse melanoma prognosis (15–18). Studies that were not able to reproduce these results have attributed the difference to confounding variables, such as UV radiation and polymorphisms in VDR (15, 19).

**Figure 1:**
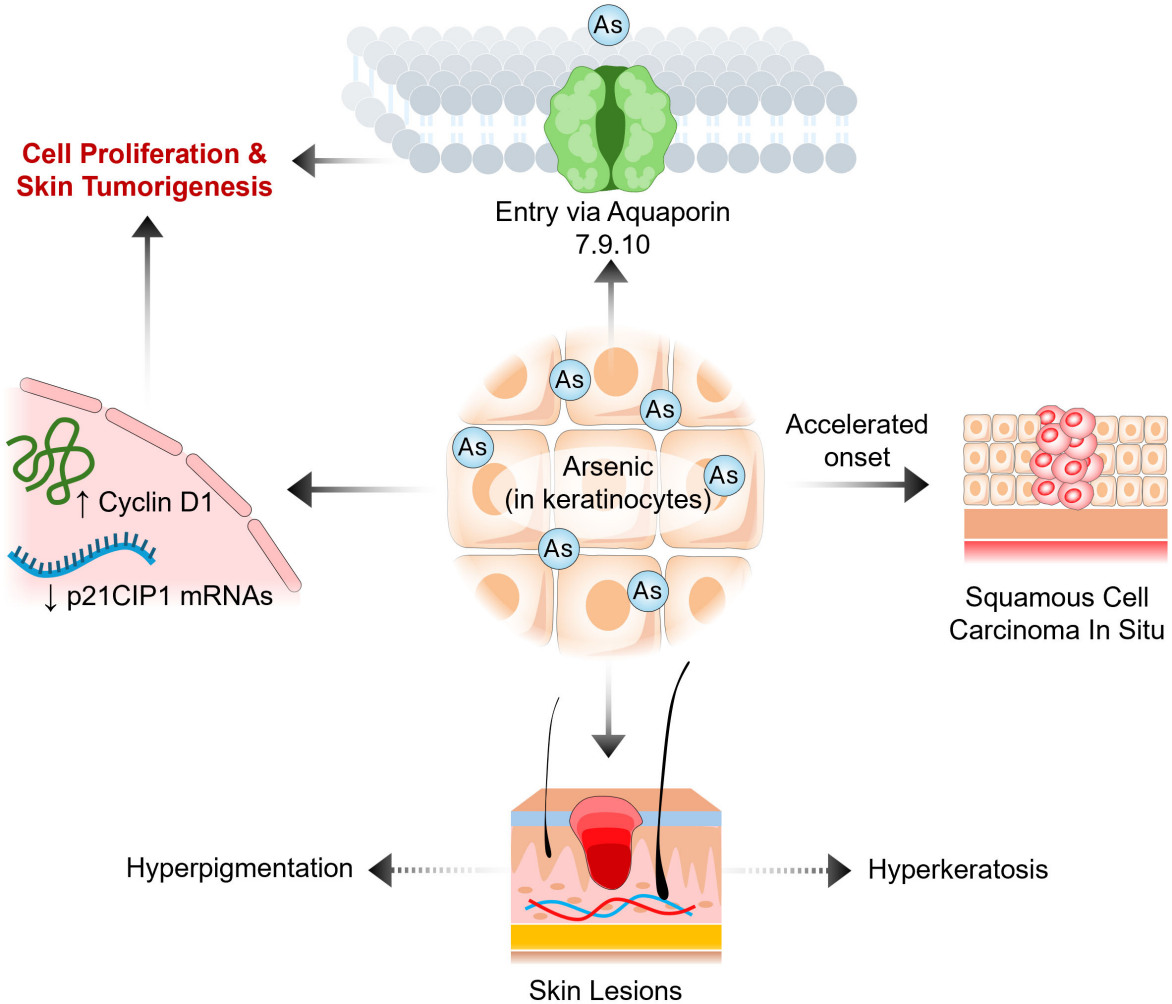
Potential events of arsenic-induced tumorigenesis of skin.

Arsenic has a unique effect on cancer; it can accelerate and decelerate the onset of cancer. The most common arsenic-induced skin cancer is squamous cell carcinoma *in situ* (20) (**Figure 1**). Arsenic is also associated with breast, lung, kidney, and liver cancers (21, 22). In fact, the International Agency for Research on Cancer classifies arsenic as a group 1 carcinogen due to its ability to cause lung cancer. In a nine-year longitudinal study, researchers determined the latency period between arsenic exposure by drinking water and the onset of cancer to be 40 years. These studies revealed that men and women have a significantly elevated risk of developing bladder cancer, with relative risks of 4.79 and 6.43, respectively, and of developing lung cancer, with relative risks of 3.38 and 2.41, respectively (22). On the other hand, numerous studies have demonstrated that arsenic trioxide can maintain remission in acute promyelocytic leukemia (APL) patients, has a low side effect profile in phase II and III clinical trials, and effectively treats APL in conjunction with chemotherapy in newly diagnosed APL patients (23). Recently, researchers have revealed a negative correlation between arsenic exposure by drinking water and the incidence of leukemia and lymphoma in men and women (24). These data demonstrate that arsenic has two different effects on hematological and solid cancers.

**Figure 2:**
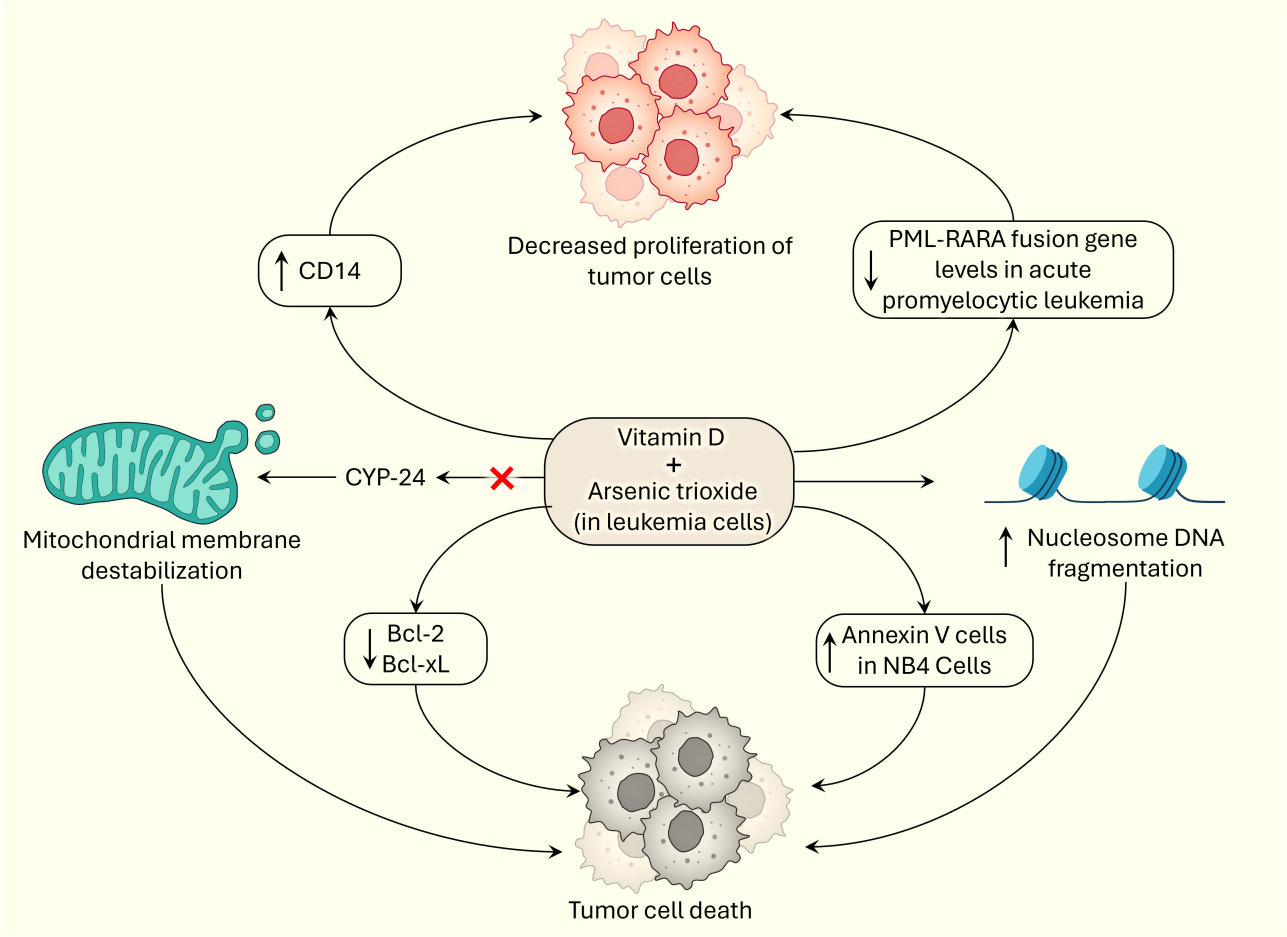
Synergistic effects of vitamin D and arsenic on tumorigenesis by inducing cell death and exerting anti-proliferative effects.

For the past decade and a half, researchers have shown that vitamin D and arsenic have synergistic effects on halting tumorigenesis and promoting apoptosis in cancer cells (**Figure 2**). In 2005, Kumagai and colleagues demonstrated that the combination of paricalcitol and As_2_O_3_ had antiproliferative effects on myeloid leukemia cells *in vitro*, especially in the HL-60 and NB-4 cell lines. Overall, paricalcitol and As_2_O_3_ increased CD14 expression, increased apoptosis, and significantly decreased the expression of the antiapoptotic proteins Bcl-2 and Bcl-xL in HL-60 cells. Kumagai and colleagues suggested that As_2_O_3_ inhibits CYP-24, which destabilizes the mitochondrial membrane and triggers apoptosis, and demonstrated how the mechanism of action of As_2_O_3_ and paricalcitol relies on phosphorylation of the ERK pathway. Their study also showed that the combination of As_2_O_3_ and paricalcitol decreased PML-RARA fusion protein levels in acute promyelocytic leukemia cells, but the underlying mechanism is unknown (25). The outcomes of this study are important because they could treat myeloid leukemias without adding to the side effect profile of traditional cancer therapies. In hematological malignancies, it has been repeatedly shown that arsenic trioxide and vitamin D synergistically induce apoptosis in tumor cells.

Rogers et al. also confirmed the cytotoxic, apoptotic, and synergistic effects of vitamin D3, rather than paricalcitol, and arsenic trioxide in *in vitro* HL-60 cells, a subtype of acute promyelocytic leukemia cells. They demonstrated apoptosis in HL-60 cells by displaying a significant increase in annexin V cells, apoptotic cells, and significant nucleosome DNA fragmentation, which is characteristic evidence of apoptosis in cells. It is hypothesized that vitamin D3 and As_2_O_3_ participate in the externalization of phosphatidylserine and nucleosomal DNA fragmentation during apoptosis. Lashkari et al. showed that vitamin D3 and As_2_O_3_ have synergistic, cytotoxic, and apoptotic effects in a time- and dose-dependent manner and significantly increase the BAX/BCL-2 ratio, specifically increasing BAX and reducing BCL-2 expression, further supporting the conclusions of Kumagai and colleagues (26); the investigators also showed a significant increase in Annexin V-positive NB4 cells, confirming the results of a study performed by Rogers et al. (27). These studies are encouraging because they further demonstrate the potential benefit of including arsenic trioxide and vitamin D in the treatment of hematological malignancies.

Unlike in hematological malignancies, arsenic may induce tumorigenesis in keratinocytes in individuals with deficient serum vitamin D, and sufficient levels of vitamin D were shown to reduce arsenic-induced tumorigenesis. In HaCaT skin keratinocytes, Yajima et al. showed that calcitriol inhibited arsenic-related anchorage-independent growth and decreased phosphorylation of the MEK, ERK1/2 and AKT pathways, which disagrees with the findings of Kumagai et al. Calcitriol also significantly downregulated Cyclin D1 and significantly upregulated p21CIP1 mRNAs; the opposite was true in arsenic-treated HaCaT cells. They also demonstrated that calcitriol significantly reduced arsenic uptake into HaCaT skin keratinocytes by halting the expression of the aquaporin genes AQP7, 9 and 10. Previous research has shown that arsenic can enter cells via Aquaporin 7 and 9 (28, 29). The calcitriol-treated cells had significantly reduced levels of intracellular arsenic, as measured by a plasma mass spectrophotometer. VDR expression significantly increased due to arsenic, but no significant change was detected with calcitriol. Yajima et al. proposed that calcitriol suppresses the expression of aquaporin genes, thereby preventing arsenic uptake and resulting in a reduction in arsenic-mediated tumorigenesis in skin keratinocytes (29). Within safe limits of sunlight exposure, UV can increase serum vitamin D levels and possibly reduce the occurrence of squamous cell carcinoma (14, 15). In conclusion, the relationship between arsenic and vitamin D is complex; in hematological malignancies, the two agents have been repeatedly shown to have synergistic effects on suppressing tumorigenesis. However, in keratinocytes, arsenic can accelerate the onset of squamous cell carcinoma. These studies illustrate that arsenic has different mechanisms of action in various cell types. More experimental studies need to be performed to further explain how arsenic, associated with vitamin D, affects other types of cancers, such as colorectal, prostate, and breast cancer.

## Vitamin D, arsenic and T-cell functions

Vitamin D deficiency and arsenic have negative effects on T-cell function. Vitamin D deficiency is linked to Crohn’s disease, multiple sclerosis, rheumatoid arthritis, and systemic lupus erythematous, all of which involve T-cell pathology (30–32). Several studies have demonstrated that inadequate serum vitamin D results in overactivation of the Th1-mediated inflammatory response, and this mechanism is primarily how Th1 cells attack pancreatic beta cells in type 1 diabetes mellitus (33). Many studies have shown that arsenic depresses T-cell function. Several studies have confirmed that arsenic alters the expression of regulatory molecules and cytokine production and causes oxidative stress in T cells (34). Specifically, a cross-sectional study showed that individuals with arsenic-induced skin lesions had a significant reduction in the secretion of the cytokines IL-2, IL-4, and IL-5 by Th1/Th2 cells (35). Others have shown that arsenic inhibits T-cell proliferation (36). *In vivo* mouse models demonstrated that arsenic alters the mRNA expression of cytokines, causing an altered ratio of Th1/Th2/Th17 differentiation in the lungs and spleen (37). Chronic exposure to arsenic and vitamin D deficiency results in immunosuppression and immunotoxicity.

In addition to tumorigenesis, arsenic and vitamin D together affect immunity and helper T-cell (Th17) function. Burchiel et al. showed that elevated urinary arsenic and its metabolites, monomethylarsonic acid and dimethylarsinic acid, were associated with a decrease in anti-CD3/CD28-stimulated T-cell proliferation in smoking males. This association still existed, but was weaker, in nonsmoking males and not in females due to confounding variables such as lower mean serum vitamin D levels, less sun exposure due to occupation, and a small sample size of smoking females. They also state that males generally had higher serum vitamin D concentrations than females and attribute this difference to sun exposure, gender occupation differences and social norms, but both men and women met the criteria of having adequate vitamin D serum concentrations of >20ng/ml. In smoking men with high serum vitamin D, T-cell proliferation and urinary arsenic showed no correlation, illustrating the protective effect of vitamin D (38). The literature reports that vitamin D and CD28 expression are linked (39); it has been shown that the VDR binding regions are close to the promoter regions of CD28 and are likely direct targets of vitamin D gene transcription (38, 40). Researchers attribute the variation in T-cell activity and proliferation to VDR polymorphisms (26, 41). Parvez et al. showed that elevated urinary arsenic and its metabolites in combination with low vitamin D (less than 20 ng/ml) and sufficient levels of vitamin D (approximately 20 ng/ml) were associated with a decrease in the number of T17 cells, but only high levels had a protective effect against arsenic and Th17 cell count. We confirmed the correlation of elevated urinary arsenic and its metabolites with lower serum vitamin D levels. Parvez et al. suspected that arsenic-related oxidative stress inhibits Lck and Fyn protein tyrosine kinase (PTK) activity, causing a decrease in Th17 cell differentiation (42–45).

## Vitamin D, arsenic, type 2 diabetes mellitus and lung functions

Vitamin D deficiency has been repeatedly shown to be linked to decreased lung capacity and function as well as type 2 diabetes mellitus. Among published studies, there are conflicting findings that show the relationship between vitamin D deficiency and lung function. One study showed a significant decrease in forced expiratory volume in one second (FEV_1_) and forced vital capacity (FVC) in individuals diagnosed with chronic obstructive pulmonary disease and a serum vitamin D concentration less than 20 ng/mL compared to those with chronic obstructive pulmonary disease alone, and confounding variables were controlled for (46–50). On the other hand, other studies have shown no significant change between serum vitamin D and FEV_1_ or FVC (51, 52), which is likely due to comorbidities. Possible mechanisms responsible for improved lung function with vitamin D sufficiency include airway remodeling (53) and immune dysregulation (54, 55). Nevertheless, there are conflicting data on the relationships among vitamin D deficiency, insulin resistance and type 2 diabetes mellitus. Both a case−control study and a meta-analysis revealed a significant correlation between low vitamin D and impaired fasting glucose levels in type 2 diabetes mellitus patients, and these findings can be attributed to how low vitamin D and elevated blood sugars together maximize total cholesterol and low-density lipoprotein cholesterol and high-density lipoprotein (46, 56). In contrast, another randomized controlled study and another meta-analysis showed that long-term daily vitamin D supplementation did not prevent the onset of type 2 diabetes mellitus (57, 58). In a prospective cohort study, McCarthy et al. showed that vitamin D deficiency increased the incidence of prediabetes, neither confirming nor denying the relationship between vitamin D and type 2 diabetes mellitus (59).

Arsenic exposure is known to have a positive correlation with diminished lung function and could be associated with type 2 diabetes mellitus. Several studies from different populations have repeatedly shown that arsenic is correlated with restrictive lung disease in humans (60). Specifically, a prospective cohort study demonstrated that arsenic at low and moderate doses significantly reduced the FEV_1_ and FEV in men, women, smokers, and nonsmokers (4). The same significant trend was observed in human fetuses who were exposed to arsenic *in utero* and whose FEV_1_ and FEV were measured at a median age of 7.4 years (61). It is unclear whether arsenic can cause type 2 diabetes mellitus in humans because much of the experimental data supporting this relationship is from mouse models *in vitro*. Arsenic exposure in laboratory rodents results in impaired glucose uptake, pancreatic β-cell defects, impaired adipocyte differentiation, and abnormal microRNA expression of several genes related to insulin secretion (62, 63). A cross-sectional human study showed a significant time-dependent and dose-dependent relationship between inorganic arsenic exposure in drinking water and the risk of developing type 2 diabetes mellitus (64).

Urinary arsenic was measured as micrograms of arsenic per gram of creatine (μg/g creatinine), and serum vitamin D was measured in nmol/L. Le et al. showed that subjects with vitamin D in the 1st quartile, having serum vitamin D levels less than 30.02 units, and urinary arsenic in the 4th quartile, having arsenic concentrations greater than 159.4 8 units, had a 302% increased risk of developing type 2 diabetes mellitus in comparison to subjects with a serum vitamin D level in the 4th quartile, greater than 55.99 units, and urinary arsenic in the 1st quartile, less than 69.67 units (65). This study demonstrated that the combination of vitamin D deficiency and elevated arsenic exposure puts individuals at increased risk for type 2 diabetes mellitus. This is important to investigate further since large portions of the world’s population are exposed to arsenic and deficient in vitamin D. Parvez et al. studied chronic exposure to arsenic in water in smoking men and showed a significant decrease in FEV_1_ and FVC. Among men who smoked and did not smoke, arsenic exposure significantly decreased the FVC but did not significantly change the FEV1. Subjects with adequate and high levels of serum vitamin D, with the same amount of arsenic exposure, had better FVC and FEV_1_ (4, 45). This study suggested that vitamin D has a protective effect on pulmonary function against chronic arsenic exposure by drinking water.

## Conclusion

In conclusion, the intricate interplay between vitamin D and arsenic has significant implications for human health, particularly in the realms of tumorigenesis, T-cell function, lung health, and type 2 diabetes mellitus. The synergy observed between vitamin D and arsenic in inhibiting hematological malignancies suggests a potential avenue for therapeutic interventions. The role of arsenic in inducing tumorigenesis requires further investigation. Vitamin D seems to play a protective role that preserves T-cell function, proliferation, and differentiation against arsenic exposure and toxicity. Additionally, this article discusses the relationship between vitamin D deficiency, arsenic exposure, and the risk of developing type 2 diabetes mellitus, emphasizing the need for further investigation. Overall, the findings discussed in this manuscript suggest that arsenic can be therapeutic in some cells but harmful in others. However, these findings prompt more studies on how arsenic affects specific groups of human cells and the clinical implications of this effect on human health. It will also be important to study the potential benefits of safe sunlight exposure for individuals with arsenic poisoning (66).

## Author contributions

MC: Writing – original draft. CU: Writing – review & editing. DB: Writing – review & editing. MR: Supervision, Conceptualization, Writing – review & editing.
